# State laws and policies to reduce opioid-related harm: A qualitative assessment of PDMPs and naloxone programs in ten U.S. States

**DOI:** 10.1016/j.pmedr.2018.12.014

**Published:** 2018-12-30

**Authors:** Christine C. Whitmore, Mary N. White, Melinda B. Buntin, Carrie E. Fry, Kevin Calamari, Stephen W. Patrick

**Affiliations:** a Department of Health Policy, Vanderbilt University School of Medicine, 2525 West End Avenue, Suite 1200, Nashville, TN 37203, United States of America; b Vanderbilt Center for Child Health Policy, 2525 West End Ave, Suite 1200, Nashville, TN 37232, United States of America; c Department of Pediatrics, Vanderbilt University School of Medicine, 2200 Children's Way, Nashville, TN 37232, United States of America; d Mildred Stahlman Division of Neonatology, Department of Pediatrics, Vanderbilt University School of Medicine, 2200 Children's Way, Nashville, TN 37232, United States of America; e Vanderbilt University College of Arts and Sciences, 301 Kirkland Hall, Nashville, TN 37240, United States of America

**Keywords:** Opioids, State policy, Prescription drug monitoring program, PDMP, Naloxone, Qualitative research

## Abstract

As the magnitude of the opioid epidemic grew in recent years, individual states across the United States of America enacted myriad policies to address its complications. We conducted a qualitative examination of the structure, successes, and challenges of enacted state laws and policies aimed at the opioid epidemic, with an in-depth focus on prescription drug monitoring programs (PDMPs) and naloxone access efforts.

A set of 10 states (Florida, Kentucky, Massachusetts, Michigan, Missouri, New York, North Carolina, Tennessee, Washington, and West Virginia) was chosen a priori to achieve a varied sample of state policies and timing, as well as population opioid complications. Archival research was conducted to identify state-level policies aimed at the opioid epidemic and semi-structured interviews were conducted with 31 key stakeholders between March and September 2016.

The most frequently mentioned key to success was an identifiable champion instrumental in leading the passage of these policies. The lack of a unified legislature and physician pushback were challenges many states faced in implementing policies.

Champion-led task forces, stakeholders' personal stories garnering buy-in, ongoing education and feedback to PDMP users, and inclusive stakeholder engagement are critical aspects of passing and implementing state policies aimed at combating the opioid epidemic. Engaging all interested stakeholders and providing continuing feedback are ongoing challenges in all states. Leveraging stakeholders' personal stories of how opioids affected their lives helped propel state efforts.

## Highlights

• Champion-led task forces and stakeholder engagement were key to opioid policy passage.
• Leveraging personal stories to garner buy-in helped propel state opioid efforts.
• A disjointed state legislature presented challenges in passing state opioid policies.
• Physician pushback and technical complications challenged PDMP implementations.
• Lack of appropriated funding for naloxone kits was a challenge to implementation.

## 1 Introduction

The frequency of opioid use and its complications have increased substantially throughout the U.S. in recent years. From 1999 to 2015, the amount of opioid pain relievers prescribed per person grew three-fold in the US (Centers for Disease Control and Prevention, 2017a), reaching over 226 million prescriptions in 2015 – a rate of nearly 71 opioid prescriptions per 100 persons in the U.S. (Centers for Disease Control and Prevention, 2017b). The rise of opioid use has been associated with neonatal opioid withdrawal (Patrick et al., 2015), opioid-related hospital and emergency department utilization (Tedesco et al., 2017; Weiss et al., 2017), and overdose deaths (Manchikanti et al., 2012; Rudd et al., 2016). In 2016 alone, the economic burden of the opioid epidemic grew to almost $96 billion (Altarum, 2018) and resulted in more than 42,000 overdose deaths (U.S. Department of Health and Human Services, 2018).

States have responded to the opioid epidemic with a variety of state-level law and policy interventions (Haegerich et al., 2014), including implementing prescription drug monitoring programs (PDMPs) (Ali et al., 2017), naloxone distribution programs (Davis and Carr, 2015; Kim et al., 2009), and regulation of pain management clinics (Rutkow et al., 2017a). In particular, PDMPs, databases that collect and store information from pharmacies dispensing controlled substances, have emerged as a common state intervention (Griggs et al., 2015; Gudoski, 2015). In most states, data collected from PDMPs can be used to identify improper and potentially dangerous prescriber behaviors (e.g., prescribing excessive amounts of opioids) and patient behaviors (e.g., “doctor shopping”) (Islam and McRae, 2014).

While research has assessed effectiveness of PDMPs at the state-level (Centers for Disease Control and Prevention, 2017b; Griggs et al., 2015; Bao et al., 2016), in depth examinations of the nuances and process of opioid-related policy implementation are limited (Levey, 2018; Rutkow et al., 2017b). The objective of this paper is to characterize state-level laws and policies aimed at reducing opioid-related harms within a purposive sample of 10 states, focusing on the implementation of PDMPs and naloxone access, and provide insights into successes and challenges of legislation and implementation from these 10 states.

## 2 Methods

### 2.1 Selection of states and key informants

Ten states were selected a priori to achieve a variation in state policies aimed at opioid misuse, timing of opioid law and policy implementation, and differences in population opioid complications: Florida, Kentucky, Massachusetts, Michigan, Missouri, New York, North Carolina, Tennessee, Washington, and West Virginia. The sample was selected to reflect variation in state policy and opioid-related complications. Sample states represent the range of PDMP experience: four have decade-long established PDMPs, while one state had not yet implemented a PDMP at the time of the study. They also vary in opioid-related complications including overdose death which ranged from 5.0 to 22.3 per 100,000 population in our sample in 2013 (the most recent year of data available when the sample was selected).

We obtained contact information for key informant interviewees in each state from PDMPassist (Prescription Drug Monitoring Program Training and Technical Assistance Center, n.d.). This list was enhanced by web searches of state offices and departments responsible for the implementation of PDMP and naloxone efforts. We utilized snowball sampling as necessary to identify additional individuals in the state knowledgeable about opioid-related policy efforts.

### 2.2 Identification of state-level policies

Current state-level opioid-related laws and policies for each of the 10 states were identified by reviewing publicly available documents from the Association of State and Territorial Health Officials and Prescription Drug Abuse Policy System websites (Association of State and Territorial Health Officials, 2018; Prescription Drug Abuse Policy System, n.d.). From these compiled excerpts of state laws, state summaries of opioid-related legislation and amendments, passage dates, and policy implementation dates were created for data tables and use in the key informant interviews. Subsequent interviews allowed for verification of this information and provided additional implementation information that could not be gleaned from the laws and regulations.

### 2.3 Stakeholder interviews

A semi-structured key informant interview guide was developed and used for each interview to address several domains of law and policy passage and implementation: 1) identifying specific opioid-related legislation passed in their state (PDMP, naloxone, and additional legislation) along with passage and implementation dates for confirmation; 2) characteristics of the state's PDMP and naloxone programs; 3) perceived successes associated with policy implementation; and 4) recognizable challenges of implementing these policies. The qualitative semi-structured interview approach was used as a grounded theory emergence strategy allowing participants to articulate their experience with minimal guidance. The semi-structured interview protocol allowed themes around the success and challenges of legislation and implementation to emerge from the participants rather than asking about specific categories. We included probes about potential issues (e.g., funding) for use if necessary, but focused on “top of mind” responses.

We began key informant recruitment with state officials and PDMP administrators given the likelihood that they would have unique insight and institutional knowledge of the practical implementation challenges of PDMPs. Additional interview participants knowledgeable of state-level opioid-related legislation and implementation came from state and county health departments, state agencies, pharmacy boards, and universities. Stakeholders were directly invited via email from the principal investigator of the study to participate in an in-depth telephone interview, frequently with multiple participants from a selected state in a single interview. To encourage frank and open discussion of successes and challenges encountered, interview participants were assured of confidentiality. Therefore, we do not attribute specific qualitative themes or quotes to named individuals or states.

### 2.4 Data analysis

Each 30-minute to one-hour interview was conducted by one team member serving as interviewer with other team members taking detailed notes. The resulting notes were reviewed and edited by both the note takers and interviewer to ensure common understandings were reflected with any discrepancies resolved jointly by the interview team.

Team members coded the key informant narratives using the structure of the interview tool as the framework (see Appendix A). All responses to a particular domain were compiled and pre-identified categories drawn from probes in the interview tool used to code responses as applicable (e.g., frequency of reporting), with iterative post-coding of subcategories as appropriate. The information was then analyzed to identify commonalities, grouping them into themes within each domain. Study team members reviewed analysis sections for fidelity to the content of the interview notes. Quantifiable data from the interviews on state-specific legislation, including passage and implementation dates, and PDMP and naloxone program characteristics were managed using REDCap (Harris et al., 2009). This project was considered exempt from human subjects review by the Vanderbilt University Institutional Review Board.

## 3 Results

**Fig. 1:**
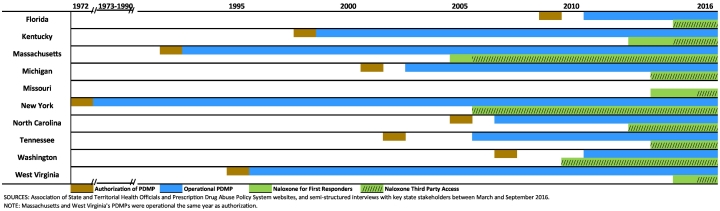
Implementation of U.S. State Prescription Drug Monitoring Programs and Naloxone Access, by Year, 1972–2016.

Between March 2016 and September 2016, we conducted 22 phone interviews with 31 key informants in the ten states. Among those contacted, we had a 96% participation rate. Two phone interviews were conducted for each state, except for Missouri (4), Michigan (3), and Massachusetts (1). To ensure confidentiality, quotes are not attributed to individuals, states, or particular roles. All sampled states, except for Missouri, had a state-wide operational PDMP at the time of data collection. As seen in Fig. 1, New York was first of the nine to have an operational PDMP (1973), and Washington and Florida most recently became operational (2011). Fig. 1 also illustrates that states' timing of implementing legislation pertaining to first responders carrying naloxone also varied.

### 3.1 Legislation passage

Interview participants were asked to identify what they considered to be successful aspects of their opioid-related law and policy passage and implementation. After analysis, several common success themes arose (Table 1), as well as challenges (Table 2) from top of mind responses.

**Table 1:** Successful features of opioid policy implementation mentioned, number of states mentioning the issue as top three, and descriptive quotes from ten study U.S. States (2016).

Success themes # States reporting issue	Descriptive quote
Engaging stakeholders 8 states	The governor in [the] state of the state [address] announced that he would hit opioid abuse head on and there was a cheer when [the governor] made that announcement.
	A lot of this was through the governor's initiative with this task force to pull everybody together and have that conversation.
	The secret sauce? Stakeholder buy-in. The problem has become so prevalent in… that you hardly meet someone who hasn't been affected.
Collaboration 8 states	[You] can't minimize the stakeholder's willingness to participate.
PDMP characteristics 5 states	A patient was in the ED with lots of pain, nothing worked for them but oxycodone. The doctor checked the PDMP and saw the patient had been prescribed 180 tablets the week before. The doctor confronted him, and the patient got up and walked out.
	Simple logic in the system, clinical decision support integrated in the system; even an office manager can see the …flags that pop up and alert a physician.
	[Providers] have personal stories that they suspect someone, and they find out later that the patient had 5 or 6 prescriptions for opioids already.
	I would have hung my hat on a particular patient's behavior and I was shocked when I checked the PDMP.
	[Funding was] not contested. Everyone understands the need.

**Table 2:** Opioid policy implementation challenges mentioned, number of states mentioning issue as top three, and descriptive quotes from ten study U.S. States (2016).

Challenge themes # States reporting issue	Descriptive quote
Lack of coordinated efforts 5 states	The legislatures may get naloxone fatigued in some ways in the fact that they have so many of these bills coming at them that they are getting confused on what each of them does and there is no coordinated effort.
Group push back 4 states	It was one more thing that they [physicians] did not want to be required to do. Their staff was too busy. It is a cumbersome process.
	The… society strongly came out against mandatory use saying I would slow down their physicians too much and maybe have a chilling effect on prescribing.
Liability concerns 4 states	Liability is the excuse for law enforcement, despite the clarity of the statute that provides immunity from all liability.
	A lot of prescribers really wanted something more concrete that says they're fully protected – that they're not going to be held liable.
Communication 3 states	The challenge is that our law enforcement partners – state police, DEA, or the Feds – sometimes have a hard time understanding that we can't just hand over everything that we may have.
Funding for naloxone 3 states	Do I buy the [naloxone] kits or do the ad campaign?
	There was no fiscal note on the bill, no additional funding. Communities and agencies had to find ways to fund these programs themselves.
	Funding continues to be a challenge – as they expand, people want more and more.
	Everyone is on board with the concept of naloxone use. Where the rubber hits the road is who is going to pay for it and how that funding will be sustained, and where the liability issue stands.
Technical challenges 7 states	[Practitioners] want it all integrated so they can click a button and see the report
	They want the doctors to do mandatory checks of the prescription system; the doctors don't want to do that because it takes a few extra minutes and the system sucks right now according to them.
	The time it takes to run a report has been a complaint – from the time you click submit to the time the report shows up on the computer screen can be a little lengthy depending on how many users are accessing the system.
	The pharmacists aren't given external emails, so it's difficult to get email to chain pharmacy staff.
Rise of illicit opioid use 4 states	The better PDMPs get, the more the heroin death rate goes up.
	As we have… reduced the opioid supply, we're continuing to see the problem become more dynamic and evolve. [Heroin and fentanyl-laced heroin] is a business proposition for people at the other end of this. [That is] different than disease vectors.

#### 3.1.1 Engagement of stakeholders

According to participants, engagement of stakeholders was critical to successful implementation of state-level opioid policies. Many interviewees reported that an identifiable champion was instrumental in leading the passage of legislation and garnering the buy-in of other stakeholders. The governor was mentioned in four states as the visible champion, often making a public announcement of prioritizing fighting prescription opioid misuse. “*This was the governor's bill; [he] helped move legislation forward.*” Other champions mentioned included attorneys general, mayors, advocacy groups, and medical directors.

For many states, the champions were those who had personal stories of patients, friends, loved ones, or constituents negatively affected by opioid addiction. “*[He has been our] biggest cheerleader; [he] also has a nephew who died of a heroin overdose, [so he] understands substance abuse.*” The pervasiveness of opioid misuse and ability to connect one-on-one with stakeholders via these personal experiences was cited by many as key to gaining buy-in, particularly in crossing partisan lines in legislatures.

#### 3.1.2 Collaboration of stakeholders

In addition to having a champion, several participants were quick to mention the positive collaboration of stakeholders that made the passage and implementation of policies possible. As one participant remarked, “*Kudos to [the] state government for working together*.” In many instances, a task force was created and provided a collaborative organizing structure to bring together stakeholders from across state agencies as well as those outside the government such as pharmacy chains and law enforcement. “*There was an array of stakeholders to tackle the prescription drug abuse issue.*” In one interview, a participant commented that prescribers and dispensers willingly came to the table because “*they saw what was happening… and decided they could not continue to be part of the problem; they must be part of the solution*.”

#### 3.1.3 State legislature challenges

The lack of coordination within some state legislatures led to multiple bills being proposed but some never leaving committee. For one state, narrowly focused bills have resulted in stakeholder group push back because they had not been invited to the table or consulted. Some states reported that their legislators “*like to tinker with bills*,” requiring that systems collect and report data points, such as overdose instances, that are neither practical nor feasible by the PDMP. For some states, privacy and data confidentiality concerns voiced by legislators have kept legislation from moving forward – “*government being ‘too much of a big brother’ and confidential information going into a database*.” Participants reported that in at least one state legislative hearing, the possibility of opioid policies “*enabling drug use*” was raised.

### 3.2 PDMP characteristics

Eight states with PDMPs have made at least one system update since initial implementation to make their PDMP a more effective tool (Fig. 1). Improvements included moving to a web-based tool, requiring more frequent data reporting, and removing barriers to utilization (Table 3). States tend to follow U.S. Drug Enforcement Agency (DEA) schedules for controlled substances classification, and, at the time of the interviews, all collect data on Schedule II-IV drugs. Kentucky, Massachusetts, Michigan, North Carolina, Tennessee, and Washington also collect data on Schedule V substances. In addition, some states collect data on non-scheduled or state-mandated drugs such as Gabapentin (Massachusetts) and hemp oil extract (North Carolina).

**Table 3:** Prescription drug monitoring program updates in nine U.S. States.

State	Year	Update details
Kentucky	2005	Online provider access
	2012	Mandatory registration for DEA-registered prescribers and KY-registered pharmacists
	2013	Daily data reporting
Massachusetts	2011	Online provider access; monitoring schedules III–V
	2016	New PMP system went live; daily data reporting
Michigan	2003	Online provider access
	2011	Bi-Monthly data reporting
	2012	Daily data reporting
	2017	Total PMP system replacement/upgrade
New York	2010	Online provider access
	2012	Real-time data reporting
North Carolina	2007	Online provider access
	2008	Bi-Monthly data reporting
	2012	Weekly data reporting
	2013	Three-day data reporting
	2016	Improvements being made to the system
Tennessee	2007	Online provider access
	2012	Weekly data reporting
	2013	Mandatory registration and use by prescribers
	2016	Daily data reporting
Washington	2012	Online provider access
	2016	Daily data reporting
West Virginia	2003	Monitoring schedules II-IV
	2004	Online provider access; Got rid of 3rd party vendor; weekly data reporting
	2012	Daily data reporting

Most participants reported that finding funding for PDMPs was not a challenge. Funding sources varied from direct appropriations, to mortgage fraud settlement monies, to piecing it together from multiple sources.

#### 3.2.1 Reporting and registration

As mandated by state laws, dispensers are required to submit dispensing information on controlled substances to the PDMP within specified data collection intervals. When the selected states' PDMPs initially became operational, most required monthly data collection, whereas Kentucky and Washington were weekly, and Tennessee required reporting bi-weekly (Table 3). At of the time of data collection (late 2016), all had moved to more frequent reporting with most now requiring daily reporting, or in the case of New York, real-time reporting. Florida requires weekly reporting and North Carolina has a 72-hour reporting requirement. Working with large chain pharmacies has brought technical challenges, including duplicate records in data dumps and absence of individual pharmacist emails. Other technical challenges participants mentioned included lack of standardized electronic records and unique patient identifiers and pacing registration when large scale changes go into effect.

In the nine states with a PDMP, registration is necessary to access information contained in the PDMP database; however, not all require prescribers and dispensers to register. Florida, Michigan, New York and Washington only require dispensers to register, whereas North Carolina requires only opioid prescribers to register for the system. While registration is important, actual system use is perhaps more important (Gudoski, 2015; Haffajee et al., 2015). All states require dispensers to report controlled substance dispensing information in their PDMP. However, only four states—Kentucky, Massachusetts, New York, and Tennessee— require all prescribers to check the system prior to prescribing opioids.

#### 3.2.2 PDMP implementation successes

Three states mentioned that having the funds and technical ability to focus on building a simple, intuitive data tool or efforts to improve their PDMP were very successful. Participants also mentioned being able to meet with initially resistant groups (“*[We were] receiving pitchforks and torches at some of these meetings.*”) a year after implementation with data from the PDMP showing improvement across the state has been gratifying. They attribute the ability of physicians and regulators to easily access the data to a decrease in doctor shopping instances and an increase in provider-patient discussions. Participants report that the system has been eye-opening for some practitioners – “*I would have hung my hat on a particular patient's behavior and I was shocked when I checked the PDMP*.” Access has also prodded some patients to call health departments complaining that their provider is changing their treatment plan based on the data available in the PDMP.

#### 3.2.3 Physician resistance

While the usability of a PDMP was considered a success by some, most states mentioned that resistance from physicians was common. Most participants reported that getting practitioners to use the PDMP has been an ongoing challenge with required registration and mandatory use the crux of the issue. Physicians felt it was an added burden to have to check the PDMP each time, particularly in states where accessing the system was cumbersome. In one instance, it was reported that many provider groups reticently “*dug their heels in*” and waited until the last minute to register, creating unanticipated technical difficulties and adding pain to a process they did not want. One key informant stated that “*one of the problems is that the people prescribing [opioids] don't understand the consequences*.” In another state, physicians “*put the ownership on the pharmacies because they are [already] required under state law to the check the PDMP*.”

Most of the states we spoke with have systems that allow physician delegates to access the PDMP for reports, but not all PDMPs are able to monitor appropriate system use. Slow systems have long been a complaint of prescribers, along with a desire for the PDMP to be integrated with and available within electronic medical records.

Getting information to users about the purpose, scope, and expected use of the PDMP has also been challenging for most states. Whether it is communicating with the large number of physicians affected by the state laws to disseminate information about how the system matches dispensing information to individuals or “*trying to get prescribers to understand that the PDMP is a clinical decision support tool that is intended to be an impetus to start a conversation with the patient*,” education and outreach to physicians is an ongoing effort for most states. Communicating with agencies and stakeholders that state health departments hadn't typically worked with was also a challenge for many states. “*[We] must learn how to communicate with prescribers, dispensers, licensing boards, and professional societies.*”

### 3.3 Naloxone initiatives

New York expanded access to naloxone for third-party users in 2006, whereas West Virginia was among the more recent to expand access in 2015 (Fig. 1). Florida, Michigan, New York, North Carolina, Tennessee, and Washington granted third-party prescription access as part of their initial naloxone legislation (Fig. 1). In these cases, a layperson such as a family member can have naloxone on hand in the case of an overdose. Many states also made access to naloxone more readily available via standing orders. Under a standing order, the naloxone provider (e.g., pharmacy, or, where permitted by law, a non-traditional provider such as a syringe exchange, public health, or community program) may distribute naloxone to an individual without that person first obtaining a traditional prescription. At the time of our interviews, all states except Michigan allowed standing orders, frequently requiring education or training before dispensing naloxone. For example, in West Virginia pharmacists must provide counseling on proper administration and the importance of contacting emergency services when dispensing naloxone. Furthermore, all ten states had a Good Samaritan law in effect at the time of the interviews, granting individuals who summon emergency responders in the event of an overdose limited immunity from prosecution for minor drug-related crimes.

That said, in some states, physicians and other prescribers have been hesitant to prescribe naloxone to third parties due to liability concerns. “*[We] heard that some in the physician community were reluctant to provide prescriptions to parents or caregivers despite the law and regulation.*”

PDMP funding has trickled in from legislative appropriation in most of the states we spoke to, with funding becoming more stable as the epidemic grew and state legislatures recognized the scope of the problem. However, funding naloxone kits has been particularly challenging. For many parents, friends, and caregivers, the kits may be covered by insurance when accessed from a pharmacy. While legislation and standing orders are in place for first responders to carry and use naloxone, funding typically wasn't appropriated to pay for the kits in the states we spoke with. Law enforcement must find their own funding to carry naloxone, with many local agencies having to find funding within their own budgets.

### 3.4 A shifting epidemic

Several states mentioned that the PDMPs have been effective in containing prescription opioids but expressed concern about a growing heroin and fentanyl epidemic as people seek opioid pain relievers from sources other than physician prescriptions, and the need to pivot to effectively address this shift. As one informant remarked, “*when you start clamping down on opioids, you see [a] diminished supply of opioids and increased price*” leading some to cheaper options such as heroin.

## 4 Discussion

With 49 state-wide PDMPs and all states implementing policies and initiatives aimed at the opioid epidemic (Haegerich et al., 2014; Compton et al., 2015), there are many implementation feature successes and challenges from which to learn. Our study of the nuances of the passage and implementation of state-level opioid-related policies adds to other recent qualitative work (Levey, 2018; Rutkow et al., 2017b) by employing a sample of ten states, examining opioid-related policies beyond PDMPs to include naloxone access, and focusing on the state-level successes and challenges of initially implementing the policies.

### 4.1 PDMPs

Previous research has assessed state opioid policy effectiveness, such as the association of PDMPs with reductions in opioid-related mortality (Nam et al., 2017; Patrick et al., 2016) and prescribing of opioids (Bao et al., 2016). Other studies suggest that the effectiveness of PDMPs lies in system design, access, and use (Ali et al., 2017; Haffajee et al., 2015; Young et al., 2017; Rutkow et al., 2015). We found that PDMPs continue to evolve to better serve stakeholder's needs. However, mandatory checking of the PDMP by prescribers is not the rule in many of the states we studied, with initial provider pushback being a significant factor. Kentucky, Massachusetts, New York, and Tennessee mandated prescriber use of the PDMP. For other states in our sample, it remains a challenge to track whether the PDMP is being utilized appropriately without requiring physician registration and mandating use (Haffajee et al., 2015). As in earlier studies (Rutkow et al., 2015; Perrone and Nelson, 2012), technical challenges remain significant barriers to PDMP use among our study sample.

### 4.2 Naloxone access

While the timely administration of naloxone has undeniably affected the opioid epidemic (Rees et al., 2017) and states and smaller jurisdictions are steadily moving to allow its use among first responders and community members (Kim et al., 2009; Bagley et al., 2018), New York was the only state in our sample to appropriate funding for naloxone. We found that many states voiced concern regarding the lack of funding, and many interviewees reported having to use a wide variety of sources for naloxone funding including grants, forfeiture funds, hospital budgets, city budgets, state budgets, and funding from local, private individuals.

### 4.3 Implementation successes

States included in our study reported the buy-in and collaboration of state government offices and agencies (Levey, 2018), a champion able to cross party lines, and sharing personal stories as keys to the success of their opioid policy implementation. Collaboration of stakeholders was a shared theme of identified successes. The pervasiveness of opioid use and ability to connect one-on-one via these personal experiences was cited by many interviewees as key to gaining legislator buy-in, particularly in crossing partisan lines in state legislatures.

### 4.4 Implementation challenges

Just as a unified legislature has been critical to successful of opioid policy implementation for states in our study, the lack of a coordinated lawmaking effort in many states has slowed, if not stopped the passage of legislation and implementation of opioid control efforts in several states. Identifying the full range of interested stakeholders, educating them, and providing continuing feedback are ongoing challenges in all states. Concern about a fluid epidemic and prescription opioid use potentially shifting toward greater use of heroin and fentanyl are also not unfounded (Dasgupta et al., 2014). There were 1960 overdose deaths attributed to heroin in 1999 and 12,989 in 2015, a six-fold increase (National Institute on Drug Abuse, 2017).

### 4.5 Limitations

Our study has important limitations. Interviews were limited to a subset of states perhaps limiting its generalizability. While we report several clear trends, they may not be representative of all state-level opioid epidemic interventions. Another limitation is the rapidly changing environment and availability of cheaper illicit opioids making it difficult for state health departments to anticipate additional legislation or new data elements necessary for their efforts to evolve with the “*moving target*” of opioid use among their populations.

## 5 Conclusion

Creating a champion-led task force and using stakeholder's personal stories to garner buy-in are reported as critical aspects of implementing policies aimed at opioid misuse, with divided legislatures and physician pushback creating the most common challenges. Involving the full range of interested stakeholders, educating them, and providing continuing feedback as well as finding funding for naloxone are ongoing challenges in the implementation of opioid use policies. There remains a need for research to address the evolution of the epidemic to inform development of comprehensive policy solutions (Haegerich et al., 2014).

## Role of the funding source

Research reported in this publication was supported by the National Institute for Health Care Management Foundation (Buntin, Calamari, Fry, Patrick, White, Whitmore) and the U.S. National Institute on Drug Abuse, National Institutes of Health under awards number K23DA038720 and R01DA045729 (Patrick). The content is solely the responsibility of the authors and does not necessarily represent the official views of the U.S. National Institutes of Health. The sponsors had no role in the design and conduct of the study; in the collection, analysis, and interpretation of the data; or in the preparation, review, or approval of the manuscript or the decision to submit.

## Contributors

Dr. Whitmore and Ms. White had full access to all the data utilized in the study and take responsibility for the integrity of the data and accuracy of the data analysis.

Study concept and design: Patrick, Whitmore

Acquisition of data: Whitmore, White, Fry, Calamari

Interpretation of results: Whitmore, White, Patrick, Buntin

Drafting of the manuscript: Whitmore, White, Patrick

Critical revision of the manuscript for important intellectual content: Patrick, Whitmore, White, Buntin

Data analysis: Whitmore, White

Obtained funding: Patrick

Study supervision: Patrick, Buntin

All authors have reviewed and approved the final manuscript.

## Declaration of interests

None.

## Conflict of interest

No conflict declared.

## Appendix A Supplementary data

Supplementary material Image 1
