# Supplementary material for: State laws and policies to reduce opioid-related harm: A qualitative assessment of PDMPs and naloxone programs in ten U.S. States
Source: Prev Med Rep. 2018 Dec 30;13:249–55. doi: 10.1016/j.pmedr.2018.12.014 (PMC6348390; doi:10.1016/j.pmedr.2018.12.014)
Supplement: Supplementary file 1 — Supplementary material [file mmc1.docx]

**The Prescription Opioid Epidemic:**

**Understanding its Complications and the Effectiveness of State Policies**

**Key Informant Interview Instrument - [STATE]**

Thank you for taking the time to speak with us today. We would like to ask you some questions about the implementation process of legislation and policies implemented in [STATE] aimed at reducing opioid pain relief (OPR) medication misuse and its complications.

**1.** What specific legislation to combat the misuse of OPRs and its complications was implemented in your state?

- - - **prescription drug monitoring program (PDMP)**
    - **NALOXONE**
- When was the [LEGISLATION] legislation passed?
  - **PDMP**
    - Legislation passed in mm/yyyy
- What was/were the implementation date(s) of the legislation? [confirm dates here]
  - PDMP
    - Data collection began on xx/xx/xxxx “Operational” date
- How long did the implementation take?
- Was it phased? What did that phasing look like?
- How well did the implementation mirror the legislation?
  - What changed between what was legislated and what was implemented?
  - What were the reasons for these changes?
- What follow up legislation was passed to strengthen/weaken the original legislation?

**[CIRCLE BACK TO TOP FROM HERE TO GO THROUGH EACH PIECE OF SPECIFIC LEGISLATION AS APPROPRIATE]**

- - **#2** NALOXONE
- When was the [LEGISLATION] legislation passed?
  - Legislation passed in mm/yyyy
- What was/were the implementation date(s) of the legislation? [confirm dates here]
- How long did the implementation take?
- Was it phased? What did that phasing look like?
- How well did the implementation mirror the legislation?
  - What changed between what was legislated and what was implemented?
  - What were the reasons for these changes?
- What follow up legislation was passed to strengthen/weaken the original legislation?

**1a.** After PDMP and Naloxone, which other two pieces of legislation or programs are the important ones aimed at reducing opioid pain relief (OPR) medication misuse and its complications in your state?

- - **#3**
- When was the [LEGISLATION] legislation passed?
- What was/were the implementation date(s) of the legislation? [confirm dates here]
- How long did the implementation take?
- Was it phased? What did that phasing look like?
- How well did the implementation mirror the legislation?
  - What changed between what was legislated and what was implemented?
  - What were the reasons for these changes?
- What follow up legislation was passed to strengthen/weaken the original legislation?
  - **#4**
- When was the [LEGISLATION] legislation passed?
- What was/were the implementation date(s) of the legislation? [confirm dates here]
- How long did the implementation take?
- Was it phased? What did that phasing look like?
- How well did the implementation mirror the legislation?
  - What changed between what was legislated and what was implemented?
  - What were the reasons for these changes?
- What follow up legislation was passed to strengthen/weaken the original legislation?
- **2.** Has a continuing medical education (CME) piece about opioids been added to the state licensure process?
  - - Required for licensure?
    - Required as punitive?
- **3.** Thinking about the legislation and programs your state implemented to combat the misuse of OPRs and its complications, what aspects of implementation stand out to you?
  - What were the challenges?
    - [MONEY/FUNDING;
    - STAKEHOLDER BUY IN;
    - TECHNOLOGY;
    - PERSONNEL;
    - OTHER?]
  - What do you consider to be the successes?
    - [MONEY/FUNDING;
    - STAKEHOLDER BUY IN;
    - TECHNOLOGY;
    - PERSONNEL;
    - OTHER?]
  - If you had to do it over again, what would you do differently?
- If you were to advise another state on implementing similar legislation and programs…
  - What do you consider to be features of a successful or “well-run” implementation/ program?
  - What “pot holes,” “speed bumps,” or “lessons learned” would you tell them to be prepared for or be aware of?
- Are there others in your office/organization that have first-hand knowledge of the implementation process/challenges/success that we should talk with? May we have their contact information?
- Is there anything else about the implementation process in your state that we haven’t discussed yet that you’d like to mention?

Thank you very much for your time today, we appreciate you sharing your implementation experiences with us. This has been essential in helping us understand the context of [STATE]’s efforts to combat OPR misuse and its complications.
